# Trismus and oral health conditions during diagnosis of malignant oral neoplasms^☆^

**DOI:** 10.1016/j.bjorl.2019.02.004

**Published:** 2019-03-18

**Authors:** Cinthia A. Martins, Dov C. Goldenberg, Rita Narikawa, Luiz P. Kowalski

**Affiliations:** A.C. Camargo Cancer Center, São Paulo, SP, Brazil

**Keywords:** Trismus, Maximal mouth opening, Oral cancer, Edentulous, Oral health, Trismo, Abertura bucal máxima, Câncer oral, Edêntulos, Saúde bucal

## Abstract

**Introduction:**

Trismus has been considered a late complication of cancer treatment. It can occur prior to treatment, mainly caused by tumor invasion or muscle spasms induced by the presence of the tumor.

**Objective:**

In this study, we evaluated the incidence of trismus and its effect on oral health in patients with malignant neoplasms of the oral cavity before performing the cancer treatment.

**Methods:**

This review was carried out via interviews, visual clinical inspection and objective measurement of maximal mouth opening in 35 consecutive patients. Trismus was defined as a maximal mouth opening <35 mm.

**Results:**

Trismus was observed in 15 patients, with a total incidence of 42%. A high rate of tooth loss was recorded, and trismus association with tooth loss was statistically verified using the Chi-square and Fisher's exact tests, the *t*-student test and Mann–Whitney non-parametric test. All tests were performed at *p* < 0.05.

**Conclusion:**

Edentulous patients are eight times more likely to have trismus compared to patients that are partially and fully dentate. Trismus was demonstrated to be correlated with tooth loss; however other oral health conditions were not shown to be a modifying factor.

## Resumo

**Introdução:**

O trismo tem sido considerado uma complicação tardia do tratamento do câncer, pode ocorrer antes do tratamento, causado principalmente por invasão tumoral ou espasmos musculares induzidos pela presença do tumor.

**Objetivo:**

Avaliar a incidência do trismo e seu efeito sobre a saúde bucal em pacientes com neoplasias malignas da cavidade bucal antes de se submeterem ao tratamento do câncer.

**Método:**

Esta revisão foi realizada por meio de entrevistas, inspeção clínica visual e mensuração objetiva da abertura bucal máxima em 35 pacientes consecutivos. O trismo foi definido como abertura bucal máxima <35 mm.

**Resultados:**

O trismo foi observado em 15 pacientes, com uma incidência de 42%. Uma alta taxa de perda dentária foi registrada e a associação do trismo com a perda dentária foi verificada estatisticamente com os testes qui-quadrado, exato de Fisher, *t* de Student e não-paramétrico de Mann-Whitney. Todos os testes foram realizados com *p* < 0,05.

**Conclusão:**

Pacientes edêntulos são oito vezes mais propensos a ter trismo do que os pacientes parcial e totalmente dentados. O trismo demonstrou estar correlacionado com a perda dentária. Entretanto, as outras condições de saúde bucal não se mostraram um fator modificador.

## Introduction

Restricted mouth opening (trismus) may be an important symptom in patients with head and neck malignant neoplasms. Trismus may have different causes, such as tumor invasion of the masticatory muscles or temporomandibular joint, inflammation of the mucosa, radiotherapy-induced fibrosis, mouth infections, edema following surgery, or pain.^1^, ^2^, ^3^

Although trismus has been considered a late complication of cancer treatment, it can occur prior to treatment, mainly caused by tumor invasion or muscle spasms induced by the presence of the tumor.^4^

There is no consensus about the incidence of trismus, which varies from 5% and 38%. in patients with head or neck cancer among different studies. This discrepancy is in part due to the lack of uniform diagnostic criteria.^1^ Currently, the most widely accepted criterion for trismus diagnosis is a Maximal Mouth Opening (MMO) of less than 35 mm.^3^, ^5^

Trismus affects the patient's quality of life negatively. Daily activities such as chewing, phonation, and breathing may be compromised. Difficulties in maintaining oral hygiene can contribute to the development of cavities, periodontitis, and other more serious dental infections.^6^

There have been few studies evaluating the incidence of trismus and its effect on oral health in oncologic patients prior to cancer treatment.^7^ The aim of the present study was to evaluate the incidence of trismus prior to cancer treatment, and analyze its relationship with subjective and objective oral health conditions.

## Methods

The present study evaluated 35 consecutive patients with malignant neoplasms of the oral cavity ICD 10 C.00 to C.08 and C14, which includes malignant neoplasms of the lip, gums, palate, floor of the mouth, mandible, parotid gland and retromolar trigone (Table 1) admitted between October 2014 and June 2015, who received no treatment before inclusion in the study.

**Table 1:** ICD-10 classification and site of neoplasm included in the sample.

Patients	ICD-10	Site of neoplasm
1	C02	Floor of the mouth e tongue
2	C060	Floor of the mouth
3	C14	Floor of the mouth, tongue, mandible
4	C03	Alveolar ridge
5	C03	Gun
6	C01	Tongue
7	C14	Mandible
8	C05	Palate
9	C02	Tongue
10	C02	Tongue
11	C02	Tongue
12	C00	Lips
13	C02	Tongue
14	C06	Retromolar trigone
15	C02	Tongue
16	C04	Floor of the mouth
17	C02	Mandible
18	C02	Tongue
19	C02	Tongue
20	C01	Tongue
21	C03	Gum
22	C05	Palate
23	C06	Retromolar trigone
24	C02	Rebordo gengival
25	C02	Tongue
26	C03	Gum
27	C00	Lips
28	C06	Retromolar trigone
29	C05	Palato mole
30	C08	Parotid
31	C06	Retromolar trigone
32	C06	Retromolar trigone
33	C02	Tongue
34	C05	Palate
35	C02	Tongue and floor of the mouth

Patients with prior history of treatment for malignant neoplasms of the oral cavity, tumor-unrelated trismus, prior trismus treatment, or who had undergone surgery of the oral cavity less than six months before were excluded from the study. Thus, we prioritize patients who never received any type of oncologic treatment, be it surgical, radiotherapeutic or chemotherapeutic, so that we could isolate the trismus caused by the presence of malignant neoplasia. The present study involving diagnostic biopsy did not disqualify subjects.

Patients were identified from the daily schedule of ambulatory appointments and invited to participate voluntarily in the study. All patients signed an informed consent form, and the study was approved by the Institutional Research Ethics Committee (no. 1875/14). In addition, the parameters adopted are in accordance with the Declaration of Helsinki.

The electronic health records for each patient included were reviewed to identify previous diagnoses and treatments received by the patient. The author of the current study performed clinical evaluations via intraoral inspection. The following elements were analyzed:

1. Dental elements: presence and number of teeth in the upper and lower arches;
2. Dental cavities (caries): quantification of compromised teeth and indication for endodontic treatment when pulp exposure was detected;
3. Gingivitis and periodontitis: signs and symptoms associated with the presence of bacterial plaque, changes in gum color and border and dental mobility;^8^
4. Tongue coating and halitosis (according to the self-perception to the patient, his companions and the examiner).

The maximal mouth opening was measured using a Willis bite gauge, considering the inter-incisor distance between central incisors, and objectively evaluated according to the criteria established by Dijkstra^3^ and Scott.^5^ Trismus severity was classified according to Thomas.^9^ Since a uniform criteria for measuring patients without teeth does not exist, the distance between alveolar ridges was measured.

Statistical analysis was performed using average, standard deviation; median, minimum and maximum values were calculated for the quantitative variables, and absolute and relative frequency distribution for the qualitative variables.

The relationships between trismus occurrence and qualitative variables were analyzed using the chi-square and Fisher's exact tests, and between trismus occurrence and qualitative variables using the *t*-student test and Mann–Whitney non-parametric test.

All tests were performed at *p* < 0.05, using the SPSS 20.0.0 software.

## Results

According the intraoral physical examination dental loss was the main change observed. Only three patients presented with complete dentition, while 23 presented with partial dentitions, and nine were completely edentulous. The total number of missing teeth was 518, with a tooth loss index of 48.6% relative to the expected total number of teeth. This, therefore, represents an average of 13.6 missing teeth per patient.

The presence of dental cavities was observed in 5 patients (4.46%), and endodontic treatment due to pulp exposure was indicated in 2 patients (5.71%). Signs and clinical symptoms of gingivitis/periodontitis were observed in 11 patients (31.4%). Tongue coating was observed in 13 patients (37.14%) and halitosis in 10 patients (28.57%).

The most affected anatomic site was the tongue with 11 cases, followed by the gum and retromolar trigone (5 cases), palate (3 cases), lips, the floor of the mouth and mandible (2 cases of each) and parotid (1 case). Tumor was present in two or more sites in four cases.

**Figure 1:**
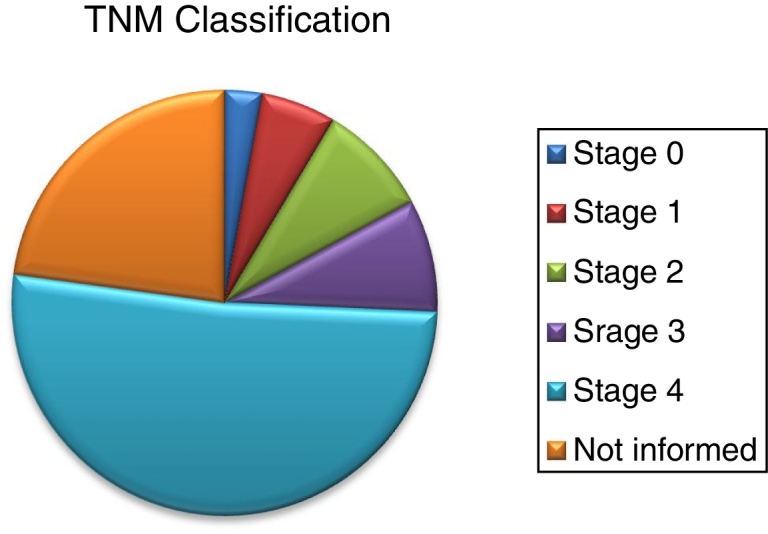
Staging of patients obtained by the TNM classification.

**Figure 2:**
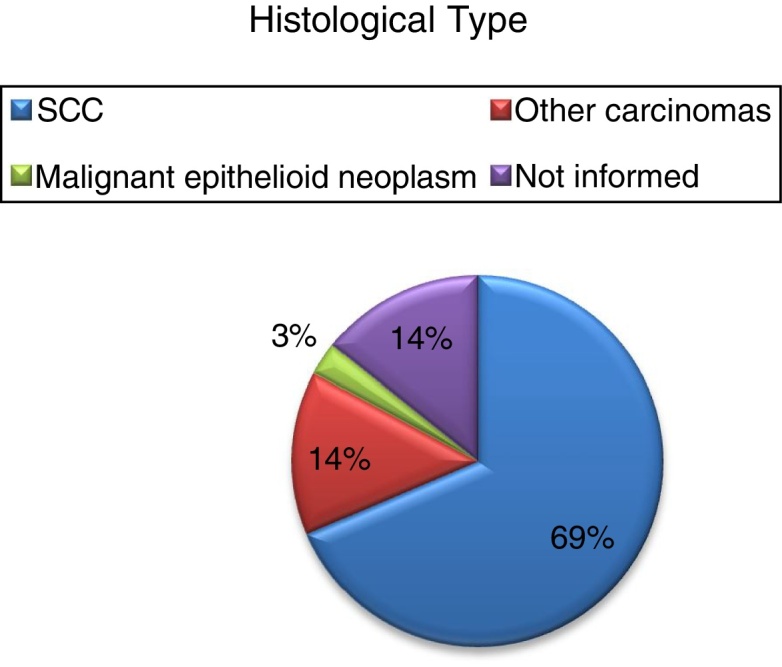
Histological type of neoplasms founded.

The most common histological type was spinocellular carcinoma (SCC), followed by other types of carcinoma and only one case of epithelioid malignancy. Tumor location as well as staging has not been shown to be a significant factor for the development of trismus, although most of the patients analyzed are in stage IV (Table 2, Table 3) (Figure 1, Figure 2).

**Table 2:** Mann–Whitney test.

Location	Trismus		Total	*p*-value
	Yes	No		
2 or more locations	2	2	4	
Gum	2	3	5	
Tongue	3	8	11	
Floor of the mouth	2	1	3	
Lip	1	1	2	
Retromolar trigone	2	2	4	0.889
Mandible	1	1	2	
Alveolar ridge	1	0	1	
Parotid	0	1	1	
Palate	1	1	2	

Total	15	20	35

**Table 3:** Descriptive analysis between neoplasm location and trismus divided into two main groups.

Location	Palate + Tongue	Others	Total	*p*-value
*n*	13	22	35	
Mean	41.846	36.023	38.186	<0.001
Median	43	34	38	
Standard deviation	9.5729	13.0004	12.0368

The analysis of mouth opening showed, at the moment of diagnosis, 15 patients with MMO less than 35 mm, corresponding to a trismus incidence of 42%. The patient distribution among trismus severity classes according to Thomas et al.^9^ is presented in Table 4.

**Table 4:** Trismus severity classification based on the criteria established by Thomas et al.^9^

Mouth Opening	Patients	Classification
<15 mm	1	Severo
15 < 30 mm	8	Moderate
30 < 35 mm	6	Mild

The overall average mouth opening for the studied patients was 38 mm. The average mouth opening for patients with trismus was 27 mm, and for patients without trismus was 46 mm. This difference was statistically significant (*p* < 0.001) (Table 5).

**Table 5:** Average MMO for patients with and without trismus.

Mouth opening	Patients with trismus	Patients wthiout trismus	Total	*p*-value
*n*	15	20	35	
Mínimum	7.5	35	7.5	
Mean	27.167	46.45	38.186	<0.001
Median	30	46.5	38	
Maxim	34	56	56	
Standard deviation	7.5868	7.0073	12.0368	

The correlation between clinical observations and trismus occurrence showed that the average number of missing teeth was 16.8 for patients with trismus and 13.3 for patients without trismus (*p* = 0.17) (Table 6).

**Table 6:** Median of number of missing teeth in patients with and without trismus.

Missing teeth	Patients with trismus	Patients without trismus	Total	*p*-value
*n*	15	20	35	
Mínimum	**0**	**0**	**0**	
Mean	16.6	11.35	13.6	0.170
Median	24	9	9	
Maxim	28	25	28	
Standard deviation	12.304	8.054	10.273	

Bold means the “highlighted” text

Edentulism incidence amongst patients with trismus was 46.66%, significantly different from patients without trismus, who presented edentulism incidence of 10%; *p* < 0.001 (Table 7).

**Table 7:** Affected teeth in patients with and without trismus.

	Affected teeth in patients with trismus	Affected teeth in patients without trismus
Missing teeth	252	266
Complete edentulous	**7**	**2**
Dental cavities	2	3
Endodontics	0	2
Teeth to be extracted	2	6

Bold means the “highlighted” text

Edentulism was possibly related to trismus occurrence prior to cancer treatment (Table 8). The chances of edentulous patients presenting trismus before cancer treatment were almost eight times higher than for patients with complete or partial dentition (OR = 7.9; 95% IC = 1.33–46.63).

**Table 8:** The relationship between edentulism and trismus.

Edentulous	Trismus		*p*-value
	Yes	No	
Yes	7	2	0.022
No	8	18	

No significant correlations among trismus and occurrence of dental cavities, gingivitis, periodontitis, tongue coating, or halitosis were observed.

## Discussion

Trismus and its associated factors deserve attention even before the beginning of cancer treatment. Although trismus is a striking factor, pre-treatment research is not performed objectively as part of diagnostic protocols. Few studies have analyzed the MMO during initial patient evaluations, and the existing studies have reported very different trismus incidence indexes.^4^, ^10^, ^11^ The lack of uniform diagnostic criteria may explain this discrepancy in the result. It is dificult when analyzing trismus incidence prior to cancer treatment, as the use of 35 mm as a threshold reflects a small limitation to normal maximal mouth opening. This is reasonable in order to guarantee higher sensitivity of diagnosis.

Considering that most of the studies evaluate mouth opening after cancer treatment, trismus diagnosis may be underestimated from an epidemiological point of view, which is in accordance with the results of the present study. Trismus diagnosis prior to cancer treatment is extremely important so that small limitations to mouth opening can be more closely monitored during the stages of surgical and radiotherapy treatments.^11^ Therefore, one of the main objectives of this study is to alert clinicians to the importance of this pre-treatment evaluation.

After surgery, mouth opening limitations, along with the presence of pain and a debilitated oral mucosa affected by neoplasia, can result in the discontinuation of oral hygiene procedures by the affected patients.^12^ The isolated effect of limitations to MMO may have a lower impact prior to surgery, when the trismus is generally less severe and not accompanied by a debilitated oral mucosa and pain secondary to surgery and radiation therapy. The association between dental morbidity and trismus may not have been established due to the large number of dental absences found in the sample, considering that the small number of teeth has a direct impact on the development of these diseases. This could explain why the presence of trismus prior to surgery had no statistically significant effect on some oral health conditions.

Overall, a considerable number of missing teeth were observed at the time of evaluation; this was attributed to the patients’ previous cancer history. Dijkstra described tooth loss as a limiting factor for the definition of the trismus criteria.^3^ The authors found no correlation between tooth loss and the development of trismus, but suggested that different cut-off points for dentate, partially dentate, and edentulous patients should be considered when establishing future criteria for trismus diagnosis. The absence of uniform criteria makes it very difficult to measure the opening of the mouth; in dentate patients the measurement is made between the incisal edges of the incisors. However, in partially edentulous patients who do not have incisors or even totally edentulous patients, it is more complicated because there is no established reference point for their measurement.

No precise cause has yet been found that explains why completely edentulous patients present a higher risk of developing trismus. Complete edentulism may indicate a history of diminished oral hygiene, and therefore delayed perception of oral problems. The delay in detecting the disease may result in its diagnosis at more advanced stages, resulting in extensive lesions to the oral cavity. In addition, edentulous patients tend to have decreased occlusal vertical dimension, which may affect the measurements and trismus diagnosis.

Trismus is a complication to cancer care that limits oral hygiene, and may result in permanent sequelae. Considering that the survival of these patients has increased greatly over the years, their early diagnosis is extremely important to ensure the maintenance of mouth opening, decrease the complexity of the treatment, and minimize the negative impact on the quality of life of cancer patients.

The sample size can be considered a limitation for the study. The specificity of the sample, as patients diagnosed with malignant neoplasms of the oral cavity and never before submitted to oncological treatments, determined a number of restricted subjects eligible to participate in the study. Also, poor oral health status may have led to a bias as to edentulism and trismus. This is an initial study of a line of research. In order to establish the real impact of edentulism on the incidence of trismus further studies should be conducted.

## Conclusion

Edentulous patients are eight times more likely to present with, trismus compared to patients that are partially and fully dentate. Trismus was demonstrated to be correlated with tooth loss; however the other oral health conditions were not shown to be a modifying factor.

## Conflicts of interest

The authors declare no conflicts of interest.
